# MRI Signal Void in Degenerated Canine Intervertebral Disks May Represent Mineralization or Gas

**DOI:** 10.1111/vru.70118

**Published:** 2025-12-19

**Authors:** Yasamin Vali, Carola Daniel, Eberhard Ludewig, Tobias Schwarz

**Affiliations:** ^1^ Diagnostic Imaging, Clinical Department of Companion Animals and Horses University of Veterinary Medicine (Vetmeduni) Vienna Austria; ^2^ Royal (Dick) School of Veterinary Studies and Roslin Institute The University of Edinburgh Roslin UK

**Keywords:** dog, gas, intervertebral disk, magnetic resonance imaging, mineralization, vacuum phenomenon

## Abstract

Accurate interpretation of intradiskal signal voids on magnetic resonance imaging (MRI) is essential to avoid misdiagnosis and improve diagnostic decision‐making in canine spinal imaging. Gas accumulation within the degenerated canine intervertebral disk (IVD), known as vacuum phenomenon (VP), and IVD mineralization are common findings in computed tomography (CT), with distinctly different x‐ray attenuation characteristics. In MRI, both appear as a signal void, which can complicate interpretation. We hypothesized that gas and mineralization would not differ in intensity values on MRI but would differ in location and shape when compared to CT. This exploratory retrospective multicenter study aims to assess the characteristics of VPs and mineralization in MRI, using CT as the gold standard. Imaging archives were searched for matching canine spinal CT and MRI performed within 48 h. Inclusion criteria included mineralization or gas in the IVD on CT images and available sagittal T2W‐sequence MRI images of the same vertebral segment. Twenty‐six studies were included, contributing 32 IVD spaces. The presence of IVD mineralization and gas in CT, along with the location, shape, and pixel‐value of MRI signal intensity in IVD spaces, was recorded. No statistical differences were identified in pixel values between the groups (*p *= 0.5). However, there were significant changes in the location of the signal void compared to the recorded location in CT, particularly in VP (*p *= 0.03). These results emphasize that MRI T2 signal voids in canine IVDs may represent gas. No specific MRI characteristics allowed reliable prioritization of VP versus mineralization; therefore, both should be considered differential diagnoses when encountering intradiskal signal voids on MRI.

**Abbreviations**
CT: computed tomography
DICOM: digital imaging and communications in medicine
IVD: intervertebral disk
MRI: magnetic resonance imaging
VP: vacuum phenomenon

## 1 Introduction

Magnetic resonance imaging (MRI) is increasingly being used as the primary imaging modality for diagnosis and treatment planning in dogs with suspected intervertebral disk disease (IVDD). For veterinary radiologists, differentiating between intradiskal gas (vacuum phenomenon, VP) and mineralization is clinically important because this distinction may influence case management, prognostic assessment, and the interpretation of degenerative pathways in canine IVDD [1]. Moreover, a clearer understanding of these MRI findings advances scientific knowledge by reducing the risk of misclassification in clinical studies and improving the accuracy of imaging‐based scoring systems [1].

The presence of gas within the intervertebral disk (IVD), known as the VP, was initially observed in humans through radiographic examinations in 1942 [2]. This finding served as an indication of degeneration within the IVD. As the degenerated disk develops small cracks, nitrogen gas is absorbed from nearby blood vessels, resulting in the formation of a gas bubble, misnamed as a vacuum [3]. This phenomenon has since been frequently observed in spinal computed tomography (CT) studies in humans and dogs with IVD degeneration [3, 4, 5]. Previously published studies have described and validated CT characteristics of gas and mineral in canine and human IVDs, including shape, location, and attenuation values [2, 4, 5]. Similarly, MRI characteristics of both mineralization and gas have been described, including artifact considerations and sequence‐dependent appearance [6, 7]. In MRI sequences, both mineralization and gas are characterized by areas of hypointensity or complete signal void [7]. Some MRI artifacts, such as the chemical shift artifact, also create areas of hypointensity [7, 8]. Differentiation of mineralization and gas in MRI has been discussed for the IVD in humans [8], but no published information could be found comparing the MRI characteristics of gas versus mineral in dogs, representing an important knowledge gap. In MRI studies of canine IVD disease, diskal hypointensities are often attributed to mineralization without consideration of the VP [9, 10, 11]. Given the high frequency of VP observed in canine spinal CT studies [12, 13], it is possible that VP is frequently present on canine spinal MRI studies but is misattributed to diskal mineralization. In dogs, diskal mineralization is associated with a specific type of IVD degeneration, called Hansen Type‐1 or IVD extrusion [1, 14], whereas VP is most frequently observed in Hansen Type‐2 or chronic IVD protrusion or fibrous metaplasia [14, 15]. The differentiation of types of canine IVD degeneration is relevant for prognosis and treatment planning [16].

The objectives of this study were to investigate whether canine IVDs with VP observed on CT exhibit diskal hypointensity or signal void areas on MRI, to characterize the specific MRI features of such potential changes, and whether hypointensities on MRI can be differentiated between gas‐ and mineral‐content‐related. Our working hypotheses were (1) that IVDs with gas do exhibit hypointensity or signal void on T2‐weighted MRI images and (2) that T2‐hypointense findings caused by mineralization or gas are indistinguishable.

## 2 Methods

### 2.1 Selection and Description of Subjects

The study was a retrospective, multicenter, and descriptive exploratory design study. Two institutions participated in the study: the Clinical Department of Small Animals and Horses, University of Veterinary Medicine (Vetmeduni), Vienna, Austria; and the Royal (Dick) School of Veterinary Studies, The University of Edinburgh, UK. Ethical approval was obtained in one institution (VERC reference 119.24, University of Edinburgh), whereas, due to the retrospective study design, no ethical approval was required in the other institution. The imaging and medical record archives of these institutions were searched for all dogs that underwent both CT and MRI of the same vertebral segment (cervical, thoracic, and lumbar) between 2018 and 2020, with a maximum 48‐h interval between the two examinations.

### 2.2 Data Recording and Analysis

**FIGURE 1:**
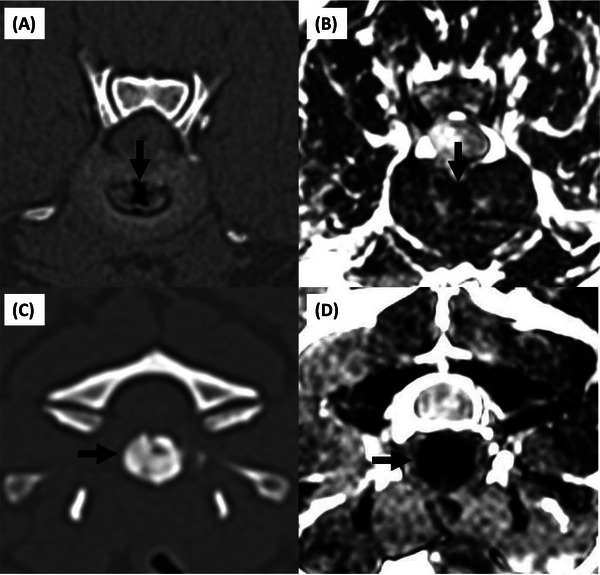
(A) Intradiskal gas (arrow) in CT and (B) corresponding intradiskal signal void on an MRI image of the same disk (arrow); (C) intradiskal mineralization in CT (arrow) and (D) corresponding intradiskal signal void on a T2‐weighted MRI image of the same disk (arrow). (A and B) The gas and corresponding intradiskal signal void are scored as “mixed shape” in the nucleus pulposus, located in Zones 1 and 5; (C and D) the mineralization and signal void are scored as “island‐shape” in the nucleus pulposus and Zone 5.

**FIGURE 2:**
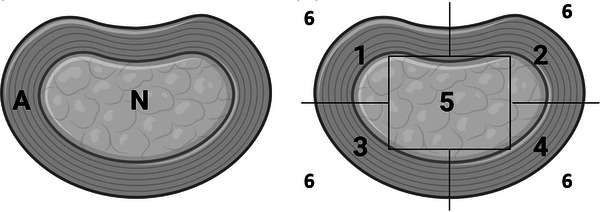
Location of the gas in CT and signal void in MRI. “N” indicates “nucleus pulposus” and “A” annulus fibrosus; 1. Right dorsal, 2. Left dorsal, 3. Right ventral, 4. Left ventral, 5. Central, and 6. Anywhere outside of the disk. *Source*: The figures were created using http://www.biorender.com.

**FIGURE 3:**
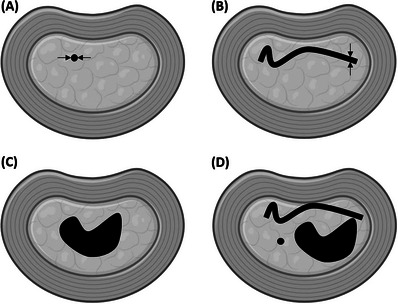
The shape of the gas in CT and signal void in MRI was classified into 4 types: (A) spot (<2 mm diameter), (B) linear (<2 mm width), (C) island, or (D) mixed. *Source*: The figures were created using http://www.biorender.com.

**FIGURE 4:**
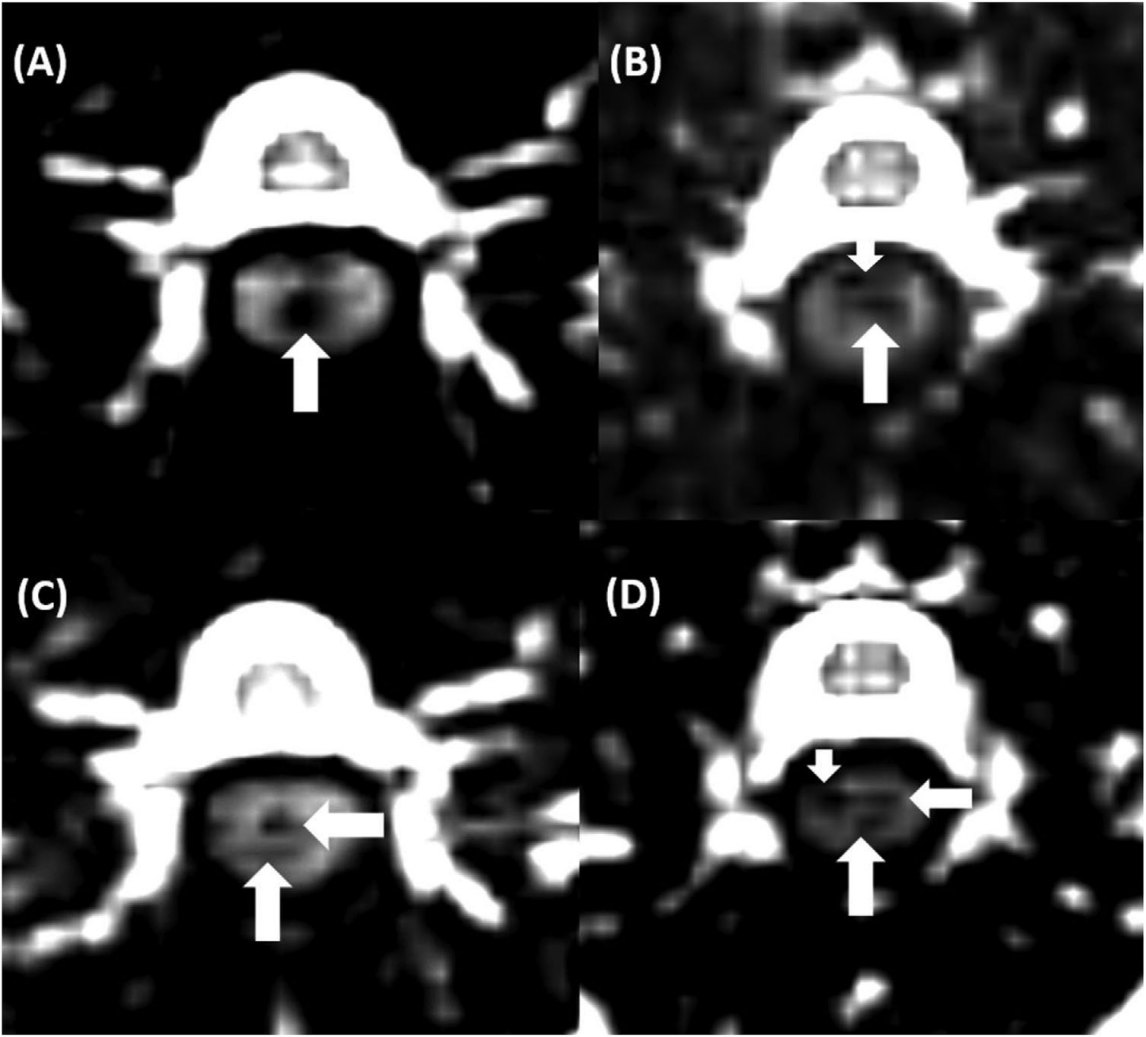
T2‐weighted MRI images showing examples of signal void areas in the intervertebral disk space. (A) Island‐shaped void in the nucleus pulposus and Zone 5; (B) linear void in the nucleus pulposus and annulus fibrosus, involving Zones 1, 2, and 5; (C) mixed shape void in the nucleus pulposus and annulus fibrosus, located in Zones 3 and 5; (D) mixed shape void in the nucleus pulposus and annulus fibrosus, affecting Zones 1, 2, and 4.

**FIGURE 5:**
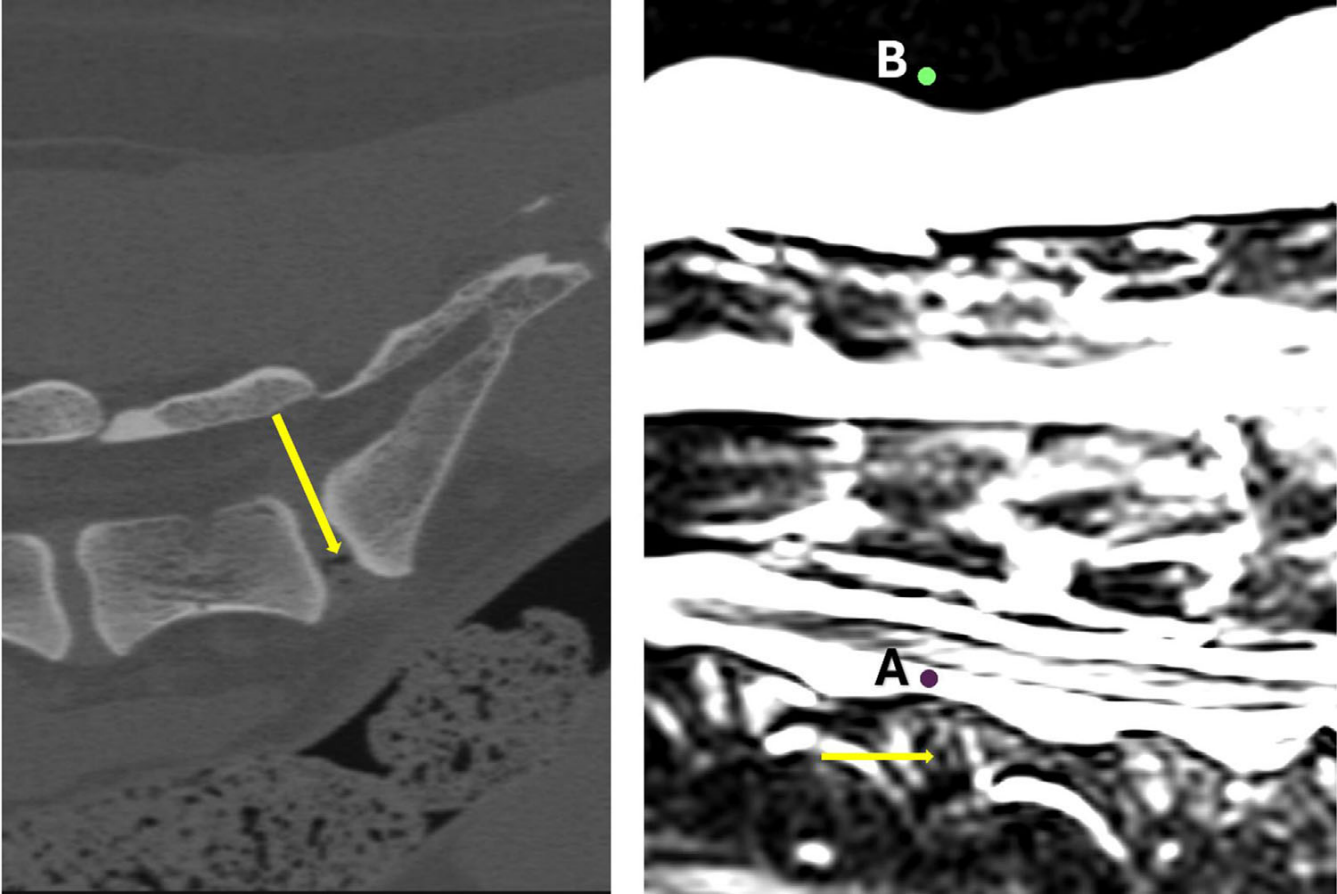
The example of two different locations for placing the point tool for measuring pixel values in the CT scan (left) and T2W sagittal sequence (right). The intervertebral gas density (left) and hypointense to signal void change (right). In both, the arrow is pointing where the measurement was done. In the right image, cerebrospinal fluid is marked as “A” and air surrounding the patient is marked as “B.” In both, the circles point to where the measurement was done.

Image analysis was performed by a third‐year veterinary radiology resident of the European College of Veterinary Diagnostic Imaging (YV) in consensus with a European board‐certified veterinary radiologist (TS, EL) using a commercial digital imaging and communications in medicine (DICOM) image viewer (Osirix_Lite, version 12.x, Pixmeo SARL, Switzerland) on a computer workstation (Imac 27‐inch, Apple, USA) with an LCD calibrated flatscreen monitor (retina display). Pairs of CT and MRI examinations were anonymized. In the first phase, anonymized CT images were reviewed for IVDs with evidence of gas or mineralization. For the purpose of analysis, IVD spaces with a change identified on CT were assigned to a gas or mineralization group. Using the point tool of the DICOM viewing software, an x‐ray attenuation value of −900 HU or less was defined as evidence of intradiskal gas and an x‐ray attenuation value of 900 HU or more as evidence of intradiskal mineralization. IVDs with both gas and mineralization were excluded from the study. A grading of intradiskal gas and mineralization was applied with a modified anatomic orientation from an established human VP grading system [1, 17]. Identified disks with gas or mineralization were graded based on location in the nucleus pulposus (N) or annulus fibrosus (A) and as (1) right dorsal, (2) left dorsal, (3) right ventral, (4) left ventral, (5) central, or (6) anywhere outside of the disk, and based on the shape as spot, linear, island, or mixed (Figures 1, 2, 3, 4). In the second phase, the anonymized MRI images were assessed by the same reviewer, non‐blinded, for the presence of intradiskal hypointense or signal void areas in the same intervertebral space as identified on CT. Using the point tool of the DICOM software on a sagittal T2 image, pixel intensity values of the hypointense to signal void change, air surrounding the patient, and cerebrospinal fluid were measured (Figure 5). Pixel intensity values are unitless, but the intensity scale is calibrated with the lowest value set at zero (black) and the highest value (white) set at 255 within an 8‐bit system [18]. A change was defined as hypointense or signal void if the pixel intensity value did not exceed 10% of the subtraction value of the air pixel intensity from the cerebrospinal fluid pixel intensity on a T2‐weighted image. For the purpose of this investigation, both hypointense and signal void findings are referred to as signal void. Identified MRI intradiskal hypointense or signal void areas were graded using the same grading system as for CT. For MRI, a T2‐weighted fast spin echo sagittal and a transverse sequence image or a three‐dimensional (3D)‐sequence covering the area of the hypointense or signal void IVD, matched to the identified disk with gas in CT, was considered minimal to be included in the study.

### 2.3 Statistics

Statistical analyses were performed using a statistical computing environment (R Version 4.2.3, R Core Team [2023] R Foundation for Statistical Computing, Vienna, Austria. URL (https://www.R‐project.org/) by a trained PhD‐level biostatistician (FR). Figures were created using a data visualization package (ggplot2: Elegant Graphics for Data Analysis Version 3.4.2.; H. Wickham. Springer‐Verlag New York, 2016).

A Fisher test was employed to assess any difference in the location and shape of intradiskal mineralized or gas changes on CT examinations. Additionally, it was used to determine whether there were changes in the location of these findings (gas or mineralization) between the CT and MRI examinations for each group, and this change was then compared between the two groups. A Mann–Whitney *U* test was utilized to assess the differences in MRI pixel value measurements between the two groups.

## 3 Results

A total of 26 studies with CT evidence of intradiskal gas or mineralization were included in the study. In 21 studies, one change, in four studies two changes, and in one study three changes were identified. In 21 studies, the CT was performed first and followed by MRI, and in five studies, MRI was performed first, followed by CT.

The CT examination was performed using a 16‐slice CT scanner (SOMATOM emotion, Siemens Healthcare, Erlangen, Germany) at the Vetmeduni of Vienna and a 64‐slice CT scanner (SOMATOM definition AS, Siemens Healthcare, Erlangen, Germany) at the University of Edinburgh. Consistent CT scan parameters used included 120 kV tube potential, 1 mm slice width, soft tissue reconstruction kernel (B40s), and helical scan mode with a collimator pitch of 0.8. The MRI examination was performed using a 1.5T MRI scanner (Magnetom Espree, Siemens Healthcare, Erlangen, Germany) using TR of 1600 ms and TE of 224 ± 3 ms, and slice thickness ranged from 0.8 to 1.5 mm, at the Vetmeduni of Vienna and a 1.5T MRI scanner (Magnetom Avanto, Siemens Healthcare, Erlangen, Germany) at the University of Edinburgh with TR ranged from 1950 to 3100 ms, TE ranged from 88 to 125 ms, and slice thickness ranged from 2.5 to 3 mm. There were 16 IVD spaces in the gas group and 16 IVD spaces in the mineralization group.

The VP group consisted of 14 dogs representing various breeds, including Labrador Retrievers (*n* = 2), Mixed‐breeds (*n* = 3), a Flat‐coated Retriever, Rhodesian Ridgeback, Spitz, Yorkshire Terrier, Cocker Spaniel, and Pointer. The breed was not specified for three dogs. In this group, the dogs were predominantly male (11 males and 3 females). The mean age of the included dogs was 114.6 months (range: 31–213 months), corresponding to approximately 9.5 years on average. In this group, the lumbosacral region (L7/S1) was the most commonly affected site (8/16), followed by L2/L3 (2/16), L1/L2, L6/L7, T9/T10, T12/T13, C7/T1, and C2/C3 (each 1/16).

The mineralization group comprised 12 dogs of various breeds, including French Bulldogs (*n* = 2), Mixed‐breeds (*n* = 3), and one representative each of Dachshund, Boxer, Shar‐Pei, Pekingese, Coton de Tuléar, and Cocker Spaniel. The breed was not specified for one dog. The dogs included 10 males and 6 females, with a mean age of 86.9 months (range: 22–172 months), corresponding to approximately 7.2 years on average. The lumbar spine was the most frequently affected region (8/16), predominantly involving L3/L4, L4/L5, L6/L7, and L7/S1, followed by the cervical (C2–C5) and thoracic (Th9–Th12) regions, which were less commonly involved.

The most commonly reported intradiskal mineralization or gas was in the nucleus pulposus. Intradiskal gas was most commonly reported within zones 3 and 5, whereas mineralization was most commonly reported in zone 5. In CT examinations, mineralization was reported more frequently than gas in the nucleus pulposus, but this was not statistically significant (*p* = 0.08). For the mineralization group, no location change between the CT and MRI examination was observed. In the gas group, there were nine location changes between the CT and MRI examination, and the difference between the CT and MRI gas location was statistically significant (*p* = 0.03). Changes in the location of intradiskal gas were reported in both annulus fibrosus and nucleus pulposus with no significant difference (*p* = 0.1921).

**FIGURE 6:**
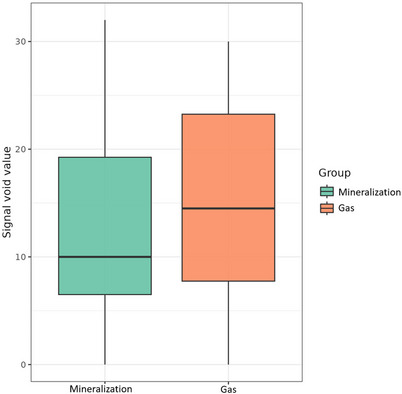
Boxplot graph indicating the distribution of the pixel values of mineralization and gas in T2‐weighted MRI images without a significant difference (*p* = 0.5).

The pixel value in MRI was measured on T2‐weighted fast spin‐echo sagittal images, acquired either in a two‐dimensional (2D) sagittal or 3D sagittal plane. No measurements were obtained from multiplanar reconstructions (MPR). The mean pixel value recorded in the MRI T2‐weighted sequence was 12.4 ± 9.27 (mean ± SD) in the mineralization group and 14.6 ± 9.72 (mean ± SD) in the gas group. No statistically significant difference was observed in pixel values between the two groups (*p* = 0.5) (Figure 6).

**FIGURE 7:**
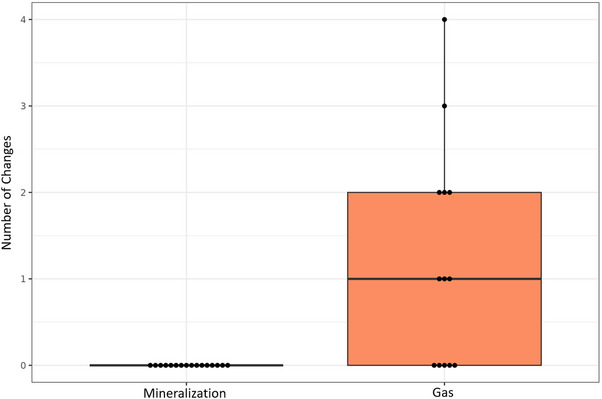
Boxplot graph indicating the number of location changes of mineralization and gas between CT and MRI examinations. Mineralization did not change location, whereas gas changed location significantly more often.

The shape of the signal void area was evaluated on T2‐weighted fast spin‐echo transverse images. Intradiskal gas and mineralization were reported most frequently in zone 5. Changes in the zonal location of gas between CT and MRI examinations were reported between zones 3, 4, and 5, with the most notable changes within the individuals in which gas was reported in zone 4 initially or subsequently (Figure 7).

**FIGURE 8:**
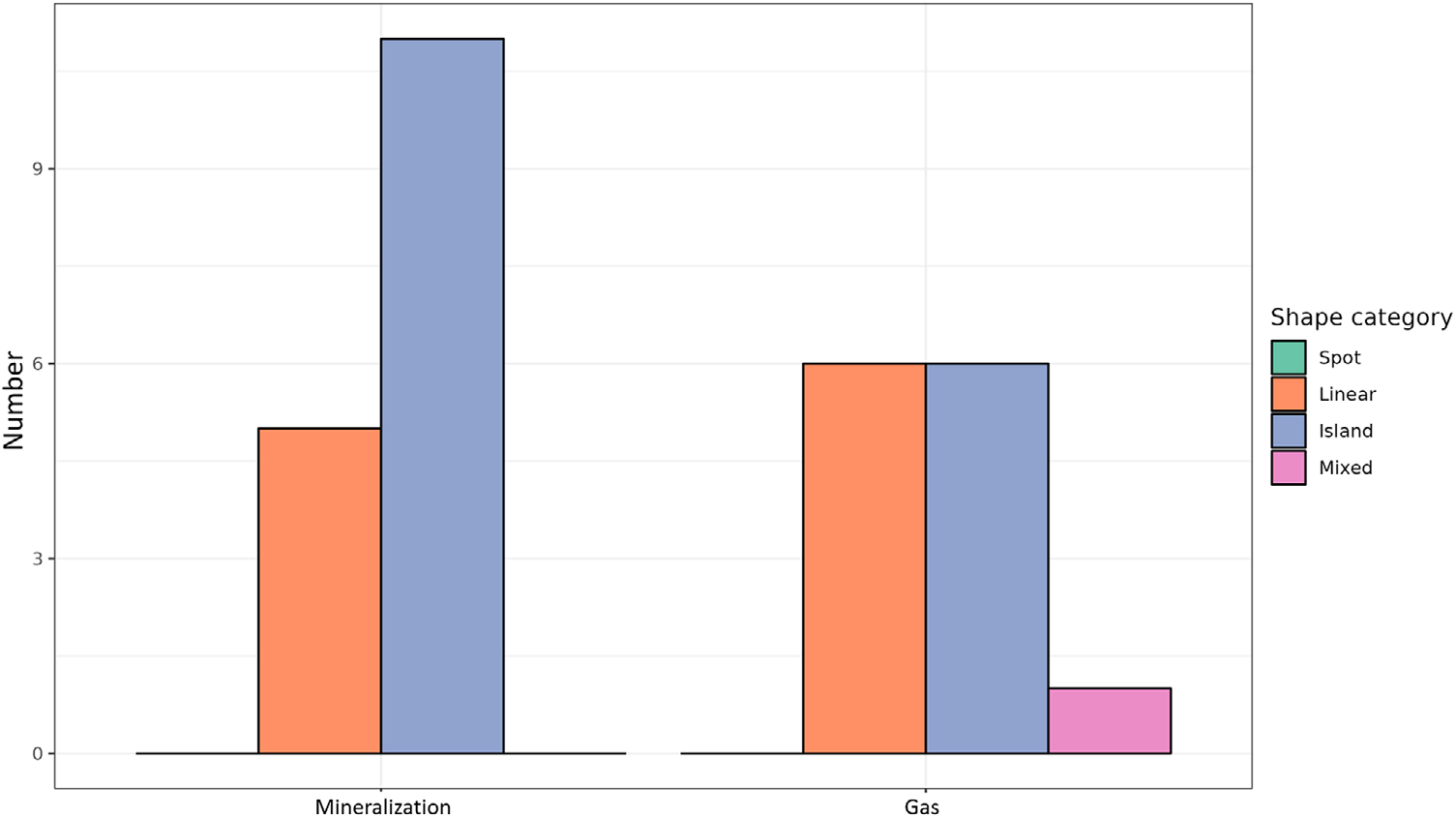
Distribution of different shapes of diskal mineralization and gas on CT images. Only in mineralization is the island shape significantly most common; only gas had a proportion of a mixed pattern shape.

**FIGURE 9:**
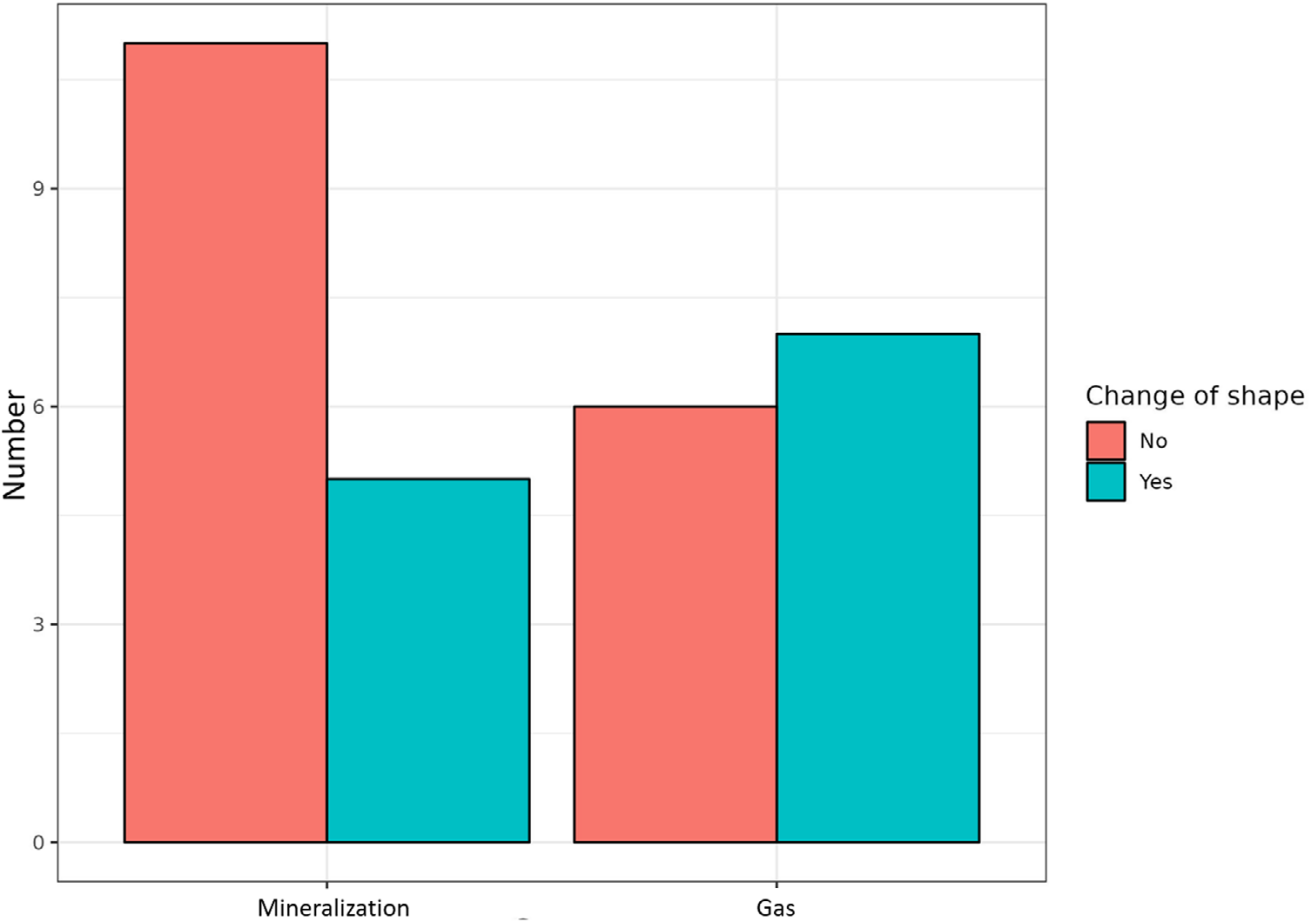
Number of shape changes of mineralization and gas between CT and MRI examinations. Gas changed shape more frequently than mineralization, but without statistical significance.

Regarding the distribution of shape categories, no spot shapes were detected in either the mineralization or gas groups in both CT and MRI, whereas the mixed pattern was solely identified in the gas group. Linear and island shapes were observed in both groups in both techniques (Figure 8). Although changes in shape categories between CT and MRI examination were reported in the mineralization and gas group, statistical analysis revealed no significant difference in the number of changes between both groups (*p = *0.2742) (Figure 9).

## 4 Discussion

This study demonstrates that IVDs with gas do exhibit hypointensity or signal void on MRI images and that these findings are indistinguishable from mineralized findings on T2‐weighted MRI images, confirming both of our working hypotheses. IVD mineralization is linked to a specific type of disk degeneration with distinct clinical signs and prognosis; however, the VP is more commonly reported in the fibrous metaplasia of the IVD [15]. It is important to consider VP and the potential for other types of degeneration when identifying MRI signal void findings in canine IVDs.

IVD disease is a comprehensive term extensively used in veterinary medicine, encompassing various changes of the IVD, including degeneration with or without herniation. In the 1950s, Hansen and Olsson significantly advanced the understanding of canine IVD disease and presented a classification system based on histopathological degenerative changes that remains influential today [14]. The classification delineates two distinct types of IVD degeneration, chondroid and fibroid metaplasia, each associated with specific breed and signalment characteristics. On the basis of this classification, canine IVD degeneration and subsequent herniation would be divided into chondroid degeneration and extrusion (Hansen Type 1) or fibroid degeneration and protrusion (Hansen Type 2) [1]. In humans, disk degeneration has been associated with VP [2]. In 1937, Magnusson described gas accumulation in human IVDs [19], which was found to be correlated to human IVD degeneration by Knutsson [2].

The most frequent reason for an intradiskal VP is disk degeneration, a process characterized by the emergence of intradiskal clefts, which are then filled with nitrogen absorbed from the blood of nearby vessels [20, 21]. These clefts gradually extend toward the annulus, displacing the central region of the disk [3, 21]. This displacement initially affects the inner fibers of the annulus fibrosus, followed by an impact on the outer fibers as the degeneration progresses, which can happen in either chondroid or fibroid degeneration and metaplasia [22]. The dystrophic calcification of the IVD occurs predominantly but not exclusively in chondrodystrophic breeds of dogs [1, 23].

The findings of the current study indicate that there is no disparity in the prevalence of intradiskal gas and mineralization within the nucleus pulposus or annulus fibrosus. This finding underscores that the specific location within the annulus or nucleus of the signal void area does not assist in determining whether this signal void is associated with mineralization or gas. Repetitive physiological flexion–extension movements can result in the gradual accumulation of gas that faces difficulty spreading backward. This leads to an elevation in intradiskal pressure until the gas is expelled through a point of lesser resistance in the annulus. The gas collects under pressure within a fibrous pseudocapsule formed by the fibers of the annulus [24].

A notable distinction was observed in the frequency of location changes between both groups, with consistent signal void locations of mineralization and frequent location changes of gas between the CT and MRI examinations. This observation aligns logically with the expectation of mineralization of fixed tissue permanently [24], whereas intradiskal gas, facilitated by the reduced resistance within the degenerated disk, is likely to move, or be absorbed and reoccur at different locations.

MRI is commonly used for the workup of spinal pathology in dogs. On MRI images, gas displays low signal intensity on both T1‐ and T2‐weighted images due to its scarcity in protons and the susceptibility artifact. This can pose challenges in distinguishing mineralization from gas, particularly in degenerated disks where water content is significantly reduced in both the annulus and the nucleus [25]. Consistent with this fundamental principle, there was no significant difference in the mean pixel intensity value recorded in the T2W MRI sequences between the mineralization and gas group in our study. VP is seen and has been reported frequently in canine spinal CT examinations [4, 15, 26]. It can be safely assumed that this gas is present in equal frequency in the canine IVD during MRI examinations. Hypointense or signal void IVD changes have been exclusively attributed to disk mineralization in canine spinal MRI publications and are reported as such in the daily practice of veterinary radiologists [4, 7]. VP should be included in the differential diagnosis of hypointense or signal void changes of the canine IVD in MRI.

In our study, both VP and mineralization were most frequently observed in zone 5 (central nucleus). It has been shown that Type 2 collagen constitutes nearly all the collagen in the nucleus in individuals up to the age of 5 years. As a part of the senile process, the percentage of Type 2 collagen in the nucleus decreases to approximately 90% of the total collagen content, which remains relatively constant in the annulus [27]. This inclination of the nucleus to be affected more and earlier than the annulus may explain the higher frequency of mineralization and gas accumulation in the nucleus identified in our study.

The assessment of VP and mineralization shapes revealed linear and island shapes in both groups, with the mixed pattern observed exclusively in VP. The absence of the spot category in MRI, despite its detection in CT, is likely attributable to limitations of MRI, resulting in a false‐negative outcome. This discrepancy may be influenced by the spatial and contrast resolution of MRI. The small size and subtle nature of the spot category might have fallen below the detection threshold of MRI, where signal voids are less conspicuous in smaller structures affected by the size of the field of view [28]. Furthermore, the ability of the observer's eye to detect small, low‐contrast changes may also play a role, as the limitations in resolution combined with subtle variations can make identification challenging. These factors highlight the challenges in identifying smaller intradiskal alterations in MRI compared to the superior contrast resolution in the detection of gas offered by CT. Changes in recorded shape were evident in both groups when comparing CT with MRI. The alteration in the shape of mineralization, detected as a signal void in MRI, is likely attributable to the susceptibility artifact, occurring in the presence of both air and mineralization [7]. Under these conditions, susceptibility differences can significantly disturb the local magnetic field, leading to a blooming artifact and focal image distortion [7]. However, alterations in the pattern of gas accumulation also seem plausible, with gas redistribution following patient repositioning between CT and MRI examinations.

This study has several limitations that should be considered when interpreting the results. First, the small number of included dogs and the large number of potential sources of variation, including dog size, partial volume averaging, imaging parameters, and scanner settings, may have contributed to the statistical results and significance. Our choice to exclude disks that contained both gas and mineral findings on CT further reduced the sample size, but we considered this important to clearly distinguish the characteristics of gas versus mineralization on MRI. Additionally, pixel values on CT and MRI reflect fundamentally different tissue properties: CT pixel values represent x‐ray attenuation relative to water [29], whereas MRI pixel values reflect the signal intensity of hydrogen protons in response to an external magnetic field and radiofrequency pulses [30]. These measures are influenced by multiple artifacts, including density changes, chemical shift artifacts, and partial volume averaging, which are different in the two methods and potentially affect the conspicuity, pixel values, and perceived shapes of intradiskal findings. Our choice to use single‐point pixel values rather than an average of multiple‐point pixel values within oval ROI's could also have increased outside sources of variability. Overcoming the limitations of the current study would be beneficial for future research. Therefore, larger sample sizes and standardized imaging protocols are recommended to minimize variability related to dog size, imaging parameters, and scanner settings. Researchers are also encouraged to utilize advanced MRI sequences with higher spatial and contrast resolution to improve the detection of subtle intradiskal changes that may fall below the MRI detection threshold. Furthermore, multi‐observer evaluations and histopathological correlation would enhance the accuracy and reliability of differentiating gas from mineralization in canine intervertebral disks in MRI.

In conclusion, hypointense or signal void areas observed in MRI within the canine IVD may arise from either mineralization or gas. This widens the differential diagnosis for the specific type of IVD degeneration. Veterinarians should be aware of this fact when interpreting canine spinal MRI studies.

## Author Contributions

**Category 1** conception and design, acquisition of data, analysis and interpretation of data: Yasamin Vali, Tobias Schwarz, Eberhard Ludewig, Carola Daniel **Category 2** drafting the article, reviewing article for intellectual content: Yasamin Vali, Tobias Schwarz, Eberhard Ludewig, Carola Daniel. **Category 3** final approval of the completed article: Yasamin Vali, Tobias Schwarz, Eberhard Ludewig, Carola Daniel. **Category 4** agreement to be accountable for all aspects of the work in ensuring that questions related to the accuracy or integrity of any part of the work are appropriately investigated and resolved: Yasamin Vali. Tobias Schwarz, Eberhard Ludewig, Carola Daniel.

## Disclosure

The authors followed Strobe‐VET network guideline disclosure. Part of the results from this article were presented as an abstract at the EVDI Annual Congress, Edinburgh, UK, September 14–17, 2022.

## Conflicts of Interest

The authors declare no conflicts of interest.

## Data Availability

The data will be made available upon reasonable request from the corresponding author.
